# Retinal Function, Risk Genotypes and Response to Nanosecond Laser Therapy in Intermediate AMD: A Pilot Study

**DOI:** 10.3390/jpm16100491

**Published:** 2026-09-23

**Authors:** Andrea Cusumano, Jacopo Sebastiani, Michele D’Ambrosio, Benedetta Regine, Raffaella Cascella, Emiliano Giardina, Benedetto Falsini

**Affiliations:** 1 Macula & Genoma Foundation, 00196 Rome, Italy; cusumano@cusumano.com (A.C.);; 2 Department of Experimental Medicine, University of Rome Tor Vergata, 00133 Rome, Italy; 3 Department of Life Science, Health, and Health Professions, Link Campus University, 00165 Rome, Italy; 4 Genomic Medicine Laboratory, LIFE Institute for Research and Health Care Santa Lucia IRCCS, 00179 Rome, Italy; emiliano.giardina@uniroma2.it; 5 Department of Biomedicine and Prevention, University of Rome Tor Vergata, 00133 Rome, Italy; 6 Macula & Genoma Foundation US, New York, NY 10017, USA; bfalsini@gmail.com

**Keywords:** age-related macular degeneration, intermediate AMD, CFH, ARMS2, electroretinography, subthreshold nanosecond laser, 2RT

## Abstract

**Purpose**: To investigate whether the major age-related macular degeneration (AMD) risk variants, *Complement Factor H* (*CFH* rs1061170, T/C) and *Age-Related Maculopathy Susceptibility 2* (*ARMS2* rs10490924, G/T), are associated with differences in full-field electroretinographic (ERG) function among patients with intermediate AMD (iAMD), and to evaluate whether retinal functional responses to subthreshold nanosecond laser (2RT^®^) therapy are influenced by these genetic variants. **Methods**: This retrospective observational study included 23 iAMD patients who underwent genotyping for rs1061170 and rs10490924 and subthreshold nanosecond laser treatment (2RT^®^). For each genetic variant, patients were stratified into those with the risk allele and those with the corresponding wild-type genotype. Full-field ERG measurements were obtained at baseline and at 1 and 3 months after 2RT^®^ treatment. Twenty-six age-matched healthy subjects served as controls for the electrophysiological comparisons. **Results**: Patients with the *CFH* risk allele showed lower photopic b-wave amplitudes than *CFH* WT/negative patients overall (group effect: *p* = 0.0431, partial η^2^ = 0.1809), whereas no corresponding group effect was observed for *ARMS2* genotypes (*p* = 0.6805). Following 2RT treatment, mixed rod–cone amplitude showed a significant effect of time (Greenhouse–Geisser-corrected *p* = 0.0355), with mean amplitudes increasing at 1 month and remaining above baseline at 3 months. Photopic b-wave amplitude also changed significantly over time in both the *CFH* (*p* = 0.0370) and *ARMS2* (*p* = 0.0342) stratifications. No significant genotype-by-time interactions were found, indicating that no genotype-dependent difference was detected in the functional response to 2RT according to *CFH* or *ARMS2* genotype. PhNR amplitude and peak time showed no significant group or time effects. **Conclusions**: The *CFH* risk allele was associated with lower photopic b-wave amplitudes, whereas no corresponding difference was observed for *ARMS2* genotypes. Following 2RT treatment, mixed rod–cone and photopic b-wave amplitudes showed significant changes over the 3-month follow-up, with no significant genotype-by-time interactions. These preliminary findings provide no evidence that the functional response to 2RT differs according to *CFH* or *ARMS2* genotypes and require confirmation in larger controlled studies.

## 1. Introduction

Age-related macular degeneration (AMD) is a progressive neurodegenerative disorder affecting the outer retina, retinal pigment epithelium (RPE), Bruch’s membrane, and choriocapillaries, and it is the leading cause of irreversible central vision loss among older individuals in developed countries [1,2]. Bruch’s membrane (BrM) is a thin, five-layered extracellular matrix located between the retinal pigment epithelium (RPE) and the choriocapillaris, providing structural support and regulating the exchange of nutrients and metabolic waste. With aging and AMD, BrM undergoes progressive thickening, lipid accumulation, calcification, and reduced permeability, impairing metabolic transport and promoting the accumulation of extracellular deposits [3]. Although aging and environmental factors contribute substantially to disease development, genetic susceptibility plays a major role in determining both disease risk and progression [4,5,6,7,8,9].

Among the numerous genetic variants associated with AMD, polymorphisms in the *Complement Factor H* (*CFH*) gene and the *ARMS2/HTRA1* locus are the strongest and most consistently replicated risk factors; CFH regulates the alternative complement pathway, with risk variants promoting complement overactivation and inflammation, whereas ARMS2/HTRA1 is associated with RPE–Bruch’s membrane/choroidal dysfunction and extracellular matrix remodeling [10,11,12,13,14,15,16,17,18]. These discoveries have established complement dysregulation, extracellular matrix remodeling, and impaired cellular homeostasis as key pathogenic pathways in AMD development and progression [13,14,19]. In addition to influencing disease susceptibility, genetic factors may contribute to phenotypic heterogeneity, affecting the clinical presentation and progression of AMD [7,8,9,15,16,17,18].

Intermediate AMD (iAMD), defined by large drusen and/or pigmentary abnormalities in the absence of choroidal neovascularization (CNV) or geographic atrophy (GA), marks a critical stage in disease progression [20,21,22]. Several therapeutic strategies have been investigated, including photobiomodulation (Valeda^®^ Light Delivery System, FDA- and EMA-approved), treatments targeting oxidative stress pathways, and subthreshold laser treatments [4,23,24,25,26,27,28], such as nanosecond laser therapy (2RT^®^). The latter has been proposed to stimulate RPE repair mechanisms and improve Bruch’s membrane homeostasis, although its precise biological effects and clinical benefits remain incompletely understood.

While structural imaging techniques such as optical coherence tomography (OCT) and fundus autofluorescence (FAF) have become central to AMD diagnosis and monitoring, functional biomarkers may provide complementary information about retinal health and disease activity. Electroretinography (ERG) offers an objective assessment of retinal function and has increasingly been recognized as a useful tool for functional phenotyping in retinal disease. Previous studies have demonstrated retinal dysfunction in AMD and have suggested possible associations between electrophysiological abnormalities and genetic risk factors. Focal ERG abnormalities have been reported in patients carrying CFH risk variants, suggesting that genetic background may influence retinal functional expression [29].

The primary aim of this study was to determine whether patients with iAMD stratified according to the major AMD susceptibility variants *CFH* rs1061170 (T/C) and *ARMS2* rs10490924 (G/T) show differences in full-field retinal electrophysiological function compared to the corresponding wild-type alleles. A secondary aim was to assess whether retinal functional responses after nanosecond laser (2RT^®^) treatment are influenced by genetic background. By combining electrophysiological assessment, genetic characterization, and longitudinal functional assessment after treatment, we sought to explore whether retinal dysfunction and treatment responsiveness in iAMD are linked to genetic susceptibility or instead reflect broader disease-related mechanisms.

## 2. Materials and Methods

### 2.1. Study Design and Participants

This retrospective observational study included 23 patients from a large cohort of AMD patients (*n* = 452) enrolled between 2015 and 2017, with intermediate age-related macular degeneration (iAMD), aged 50 years or older, who underwent genotyping and full-field ERG assessment at baseline and at 1 and 3 months. Twenty-six age-matched healthy subjects without retinal disease served as controls for the electrophysiological reference values. The mean age of controls was 69.2 (±5.4) (range, 56–82), 8 males and 18 females. For each patient, ERG data from the treated eye were included in the analysis. The best eye was chosen according to clinical and multimodal evaluation.

Patients underwent a complete ophthalmological examination, including best-corrected visual acuity (BCVA), full-field electroretinography (ERG) using Retimax (CSO, Florence, Italy) and spectral-domain OCT with Spectralis (Heidelberg Engineering, Heidelberg, Germany), and fundus autofluorescence (FAF). Patient eligibility and iAMD staging were primarily based on clinical examination and multimodal imaging findings, with OCT biomarkers representing the main imaging criteria for patient selection and disease staging.

Patients fulfilled the clinical criteria for intermediate AMD as defined by Ferris et al. Large drusen (>125 μm in diameter) and/or pigmentary abnormalities were present within 1500 μm of the foveal center [20]. Patients with evidence of neovascular AMD, geographic atrophy, reticular pseudodrusen, or other retinal disorders potentially affecting retinal function were excluded.

When both eyes fulfilled the eligibility criteria, the study eye was selected according to BCVA, with the eye showing the better BCVA being included in the analysis. This criterion was applied consistently and independently of the ERG findings in order to minimize potential selection bias related to the electrophysiological outcomes.

Clinical and multimodal imaging examinations were acquired and independently evaluated by trained technicians according to standardized protocols, under the supervision of an experienced ophthalmologist (A.C.), who confirmed the clinical diagnosis, disease staging, study-eye eligibility, and absence of exclusion criteria.

This study was approved by the Institutional Review Board (IRB) Ethics Committee of Santa Lucia Foundation IRCCS (CE/PROG.650 approved on 1 March 2018) and adhered to the tenets of the Declaration of Helsinki. Participants provided written informed consent for clinical and genetic investigations.

Demographic, clinical, and genetic characteristics of the study cohort are presented in Table 1.

### 2.2. Genetic Analysis

All participants underwent genetic counseling and provided written informed consent for genetic testing before buccal swab collection. Genomic DNA was isolated from buccal swabs [30] using the MagPurix Automatic Extraction System (Resnova, Rome, Italy) according to the manufacturer’s instructions. DNA concentration and quality were assessed by DeNovix spectrophotometer (Resnova). All samples were genotyped for the two selected variants in the *CFH* and *ARMS2* genes. Genotyping was performed using TaqMan^®^ SNP Genotyping Assays on the QuantStudio™ 5 Real-Time PCR System (Thermo Fisher Scientific, Waltham, MA, USA), according to the manufacturer’s instructions. Genotype calls were generated and reviewed using QuantStudio™ Design and Analysis Software (Thermo Fisher Scientific, version 2.7.0). Quality control included no-template negative controls and previously characterized positive control samples to ensure the accuracy and reliability of genotyping.

Genotyping call rate was 100% for both variants. Genotype frequencies for CFH rs1061170 and ARMS2 rs10490924 could not be reliably assessed for Hardy–Weinberg equilibrium given the limited sample size of this pilot cohort.

For the statistical analyses, patients were classified into two groups for each genetic variant: those with the wild-type genotype and those with at least one risk allele, including both heterozygous and homozygous individuals.

CFH rs1061170 (Y402H) and ARMS2 rs10490924 were selected a priori as the two most extensively replicated AMD susceptibility loci; this pilot study was deliberately restricted to these two proof-of-concept markers and was not designed to capture the full polygenic architecture of AMD, which also involves additional established loci (e.g., C3, CFI, CFB, APOE, TIMP3, COL8A1, VEGFA, HTRA1) and polygenic risk scores. Furthermore, the dominant genetic model adopted here (wild-type versus carriers of at least one risk allele) was chosen to preserve statistical power given the small sample size; this approach does not allow assessment of a potential dose-dependent (additive) effect of the risk allele, which should be explored in larger cohorts.

### 2.3. Subthreshold Nanosecond Laser Treatment

All patients voluntarily underwent treatment with a commercially available nanosecond laser system (2RT^®^, Ellex Medical Lasers, Adelaide, Australia).

Laser treatment consisted of twelve subthreshold applications: 6 below the superior and 6 above the inferior vascular arcades, using a 400 μm spot size and a 3-nanosecond pulse duration. Individualized test spots were used to determine the energy level required to produce a barely visible threshold lesion. Treatment was then delivered at subthreshold settings in accordance with previously published protocols [28].

### 2.4. Electroretinography

Full-field ERGs were recorded with skin electrodes according to a standardized clinical protocol based on international recommendations for full-field ERG recording and consistent with a previously published skin-electrode protocol [31].

Following pharmacological pupil dilation (1% tropicamide and 2.5% phenylephrine hydrochloride), recordings were obtained under standardized dark-adapted and light-adapted conditions.

After 30 min of dark adaptation, mixed rod–cone ERGs were elicited using full-field white flashes (0.5 cd·s/m^2^). Responses were averaged over 20 stimulus presentations.

Following 20 min of light adaptation, cone-mediated ERGs were recorded using white flashes of 2 cd·s/m^2^ presented on a steady white background of 20 cd/m^2^. Responses were averaged over 40 stimulus presentations.

Signals were amplified (10,000), filtered (0.1–200 Hz), digitized (2 KHz, 12 bit resolution), and averaged (20 sweeps) with automatic artifact rejection.

The primary electrophysiological outcome measures included the following:

- mixed rod–cone ERG amplitude;
- photopic b-wave amplitude;
- photopic b-wave peak time;
- photopic negative response (PhNR) amplitude;
- PhNR peak time.

The mixed rod–cone and cone-mediated b-wave amplitudes were considered indices of outer retinal and bipolar cell function, whereas the PhNR was used as an indicator of inner retinal activity, primarily reflecting ganglion cell and glial cell function.

### 2.5. Statistical Analysis

Patients were stratified separately according to *CFH* and *ARMS2* genotype into two groups: wild-type and individuals with at least one risk allele. Patients were also stratified according to their heterozygotes and homozygotes status. Since the study was exploratory, no a priori calculation was performed; a post hoc sensitivity analysis showed that the achieved sample had 80% power to detect between-genotype differences in d ≥ 1.23 (CFH) and d ≥ 1.26 (ARMS2), and within-subject changes in dz ≥ 0.61. Homogeneity of variance between genotype groups was assessed using Levene’s test. When this assumption was met, a mixed repeated-measures analysis of variance was performed, with genotype group as the between-subjects factor and time (baseline, 1 month, and 3 months) as the within-subjects factor. Sphericity was assessed using Mauchly’s test, and Greenhouse–Geisser-corrected *p* values were reported when required. Effect sizes were expressed as partial eta squared (partial η^2^). When homogeneity of variance was not met, between-group comparisons at each time point were performed using independent-samples *t*-tests with Welch correction, while changes over time within each group were assessed non-parametrically with Bonferroni-adjusted post hoc comparisons. Statistical significance was set at *p* < 0.05. Analyses were performed in Python (version 3.12.3) using the pingouin (version 0.6.1) and scipy libraries (version 1.17.1).

### 2.6. Follow-Up and Outcome Measures

ERG recordings were obtained at baseline (same day of the treatment) and repeated at 1 and 3 months after 2RT treatment. Although 6-month recordings were available in selected patients, the inferential longitudinal analysis was restricted to baseline, 1 month, and 3 months.

The primary outcome measure was the relationship between retinal electrophysiological function and AMD risk genotype.

Secondary outcome measures included changes in ERG parameters after subthreshold nanosecond laser treatment and their relationship to genetic background.

## 3. Results

### 3.1. Baseline Retinal Function and Genotype Analysis

Relative to the electrophysiological reference values obtained in age-matched healthy controls (Table 2), patients with intermediate AMD showed lower mean mixed rod–cone and photopic ERG amplitudes, consistent with mild generalized retinal dysfunction.

No significant differences were found between heterozygotes and homozygotes for both *CFH* and *ARMS2.* Patients were consequently stratified according to *CFH* genotype into patients with the risk allele (*n* = 11) and WT patients (*n* = 12) and according to *ARMS2* genotype into patients with the risk allele (*n* = 9) and WT patients (*n* = 14). Photopic b-wave amplitudes were lower overall in *CFH* risk allele than in *CFH* WT/negative patients (group effect: *p* = 0.0431, partial η^2^ = 0.1809). Mean photopic b-wave amplitudes in *CFH* risk allele and WT/negative patients were 15.43 ± 4.95 versus 22.16 ± 7.39 µV at baseline, 20.04 ± 5.63 versus 23.75 ± 8.75 µV at 1 month, and 18.88 ± 3.58 versus 22.81 ± 5.21 µV at 3 months, respectively.

No corresponding group effect was observed in the *ARMS2* stratification (*p* = 0.6805). Mean photopic b-wave amplitudes in *ARMS2* risk allele and WT/negative patients were 21.05 ± 7.22 versus 17.59 ± 6.92 µV at baseline, 21.68 ± 8.41 versus 22.17 ± 7.18 µV at 1 month, and 21.03 ± 4.51 versus 20.86 ± 5.20 µV at 3 months, respectively.

Mixed rod–cone amplitude, PhNR amplitude, and PhNR peak time showed no significant group effects in either genetic stratification.

In the *CFH* analysis, a nominal between-group difference in photopic b-wave peak time was observed at 1 month, with longer values in patients with *CFH* risk allele than in *CFH* WT/negative patients (35.83 ± 1.93 versus 34.19 ± 1.73 ms; Welch’s *p* = 0.045). The corresponding differences at baseline and 3 months were not significant. No significant difference in photopic b-wave peak time was observed between the *ARMS2* groups.

Representative multimodal imaging findings are shown in Figure 1.

### 3.2. Functional Response to Subthreshold Nanosecond Laser Treatment

During the 3-month follow-up after 2RT treatment, mixed rod–cone amplitude showed a significant overall effect of time in both the *CFH* and *ARMS2* stratifications (Greenhouse–Geisser-corrected *p* = 0.0355). In the *CFH* analysis, mean mixed rod–cone amplitudes in patients with *CFH* risk allele increased from 34.99 ± 16.04 µV at baseline to 44.46 ± 15.01 µV at 1 month and were 45.20 ± 11.21 µV at 3 months. In *CFH* WT/negative patients, the corresponding values were 43.28 ± 12.40, 49.39 ± 11.10, and 47.23 ± 9.27 µV. Neither the *CFH* group effect (*p* = 0.2151) nor the genotype-by-time interaction (*p* = 0.5843) was significant.

In the *ARMS2* stratification, mean mixed rod–cone amplitudes in patients with *ARMS2* risk allele were 43.78 ± 10.82 µV at baseline, 46.65 ± 13.30 µV at 1 month, and 44.00 ± 9.76 µV at 3 months. The corresponding values in *ARMS2* WT/negative patients were 36.44 ± 16.24, 47.28 ± 13.39, and 47.71 ± 10.33 µV. The *ARMS2* group effect (*p* = 0.8156) and genotype-by-time interaction (*p* = 0.1741) were not significant.

Photopic b-wave amplitude also showed a significant effect of time in both the *CFH* (*p* = 0.0370, partial η^2^ = 0.1452) and *ARMS2* (*p* = 0.0342, partial η^2^ = 0.1485) stratifications. No significant genotype-by-time interactions were observed for photopic b-wave amplitude in either the *CFH* (*p* = 0.3547) or *ARMS2* (*p* = 0.2060) analysis. PhNR amplitude and peak time showed no significant effects of time or genotype-by-time interactions.

No genotype-dependent difference in the temporal ERG response was detected during the 3-month follow-up. An illustrative individual ERG series is shown in Figure 2, while group-level longitudinal changes in mixed rod–cone and photopic b-wave amplitudes are summarized in Figure 3.

## 4. Discussion

This retrospective pilot study was designed to investigate whether the major AMD susceptibility variants rs1061170 (*CFH*) and rs10490924 (*ARMS2*) influence retinal electrophysiological function in patients with intermediate AMD and whether *CFH* and *ARMS2* genotypes affect retinal functional responses following subthreshold nanosecond laser 2RT^®^ treatment.

Three principal findings emerge from the present study.

First, patients with intermediate AMD showed evidence of mild but measurable generalized retinal dysfunction relative to the electrophysiological reference values obtained in age-matched healthy controls. Reduced mixed rod–cone and photopic ERG amplitudes suggest that retinal impairment in iAMD is not limited to localized macular abnormalities visible on structural imaging and may involve broader retinal functional alterations. These findings align with previous reports in AMD indicating that retinal functional abnormalities may not parallel clinically detectable funduscopic changes and may reveal differences not captured by conventional visual acuity or structural assessment [27,29].

Second, mixed rod–cone amplitudes did not differ significantly according to *CFH* or *ARMS2* genotypes. In contrast, photopic b-wave amplitudes were lower overall in patients with *CFH* risk allele than in *CFH* WT/negative patients, whereas no corresponding group difference was observed for *ARMS2*. PhNR amplitude and peak time were also comparable across genotype groups. These findings suggest that *CFH* genotype may be associated with differences in cone-mediated retinal function, while generalized mixed rod–cone and inner-retinal electrophysiological measures appear less strongly related to the genetic variants examined. This pattern is consistent with the previous pilot study by Capoluongo et al., which reported cone-mediated functional abnormalities in relation to AMD risk variants [29]. No definitive conclusions can be drawn for the ARMS2 genotype since only one homozygote was included in the study.

The selective involvement of the photopic b-wave also deserves consideration. Whereas the ERG a-wave predominantly reflects photoreceptor activity, the b-wave mainly reflects post-receptoral signal transmission, particularly the activity of ON bipolar cells. Both components may therefore provide relevant information in iAMD; however, in the present cohort, detectable changes were observed in b-wave amplitude, whereas no corresponding changes in a-wave amplitude were identified. This pattern may suggest that subtle functional abnormalities in post-receptoral retinal signaling are more readily detectable than alterations in the photoreceptor response itself in this setting. One possible biological interpretation is that changes in the retinal microenvironment, including inflammatory and microglial processes, could influence bipolar-cell function and thereby modulate the b-wave response. Alternatively, b-wave changes may indirectly reflect subtle alterations in photoreceptor input that were insufficient to produce detectable changes in the a-wave. These interpretations remain speculative, and the present study was not designed to determine the cellular mechanisms underlying the observed electrophysiological findings.

From a mechanistic standpoint, the association between the CFH risk allele and reduced photopic b-wave amplitude may be interpreted within the framework of complement biology: the CFH rs1061170 risk variant impairs regulation of the alternative complement pathway, which may promote chronic low-grade complement activation and subclinical inflammation at the level of the retinal pigment epithelium; the resulting RPE stress and altered photoreceptor support could in turn compromise bipolar cell signaling, providing a plausible biological rationale for the reduced photopic b-wave amplitude observed in CFH risk allele carriers. The absence of a comparable effect for ARMS2 deserves further comment and may reflect at least three, non-mutually exclusive explanations: (i) limited statistical power to detect a modest effect in this small cohort; (ii) a biological mechanism distinct from that of CFH, with ARMS2/HTRA1 more directly implicated in extracellular matrix remodeling than in complement-mediated inflammation; and (iii) a role for ARMS2 that may be more relevant to disease progression than to baseline retinal electrophysiological function. In this study, no complement measurements, and no inflammatory biomarkers were measured. Given the exploratory nature of our study, we can only suggest a hypothesis rather than a mechanistic interpretation.

The limited genotype-related differences observed in the present study do not diminish the established role of *CFH* and *ARMS2* in AMD susceptibility and progression. Rather, they suggest that the full-field ERG phenotype may be largely shared across genetic groups, with the clearest difference involving photopic b-wave amplitude in patients with the *CFH* risk allele.

The third main finding was a significant overall change in mixed rod–cone and photopic b-wave amplitudes during the 3-month follow-up after 2RT treatment. Mean amplitudes were higher at 1 month and remained above baseline at 3 months. However, given the retrospective and uncontrolled study design, these longitudinal changes cannot be attributed to a therapeutic effect of 2RT. In particular, the absence of a sham-treated or untreated control group prevents discrimination between a treatment-related effect, spontaneous or natural variability in ERG measurements, and potential effects related to repeated testing. No significant genotype-by-time interactions were detected.

These results provide no evidence that the functional response observed after 2RT differed significantly between patients according to their *CFH* or *ARMS2* genotypes. In this context, evidence from long-term follow-up studies in DHRD has shown that 2RT treatment may stabilize or even improve retinal functional parameters, supporting the hypothesis that subthreshold nanosecond laser therapy may exert beneficial effects beyond structural changes alone [32].

Although the precise mechanisms underlying this response remain incompletely understood, experimental evidence indicates that nanosecond laser treatment triggers a complex biological response involving retinal pigment epithelial remodeling, extracellular matrix turnover, matrix metalloproteinase activation, and improved transport across Bruch’s membrane [33,34,35].

From a broader biological perspective, the electrophysiological changes observed in the present study may reflect retinal resilience mechanisms activated by controlled subthreshold injury. The concept of acquired resilience has been proposed as a fundamental adaptive response in which mild cellular stress triggers endogenous protective pathways that enhance tissue function and resistance to subsequent damage [36]. Although speculative, this framework may provide a useful biological model for interpreting the observed increase in mixed rod-cone and photopic b-wave amplitudes during the 3-month follow-up after 2RT treatment.

The present study should be interpreted considering certain limitations, including its retrospective design, relatively small sample size, and limited follow-up duration. Because of the retrospective design and absence of a sham-treated or untreated control group, these observations should be considered preliminary and require confirmation in prospective controlled studies.

Given the small sample size (23 patients overall, including 11 CFH risk-allele carriers versus 12 wild-type, and 9 ARMS2 risk-allele carriers versus 14 wild-type), the present study is likely underpowered to detect modest genotype–treatment interactions; the absence of statistical significance should therefore not be interpreted as evidence of the absence of a genotype effect. Future studies with larger cohorts should consider multivariable regression models (e.g., ERG amplitude as a function of age, sex, and CFH/ARMS2 genotype) rather than simple group comparisons, formal testing of a CFH × ARMS2 interaction given reported additive effects of these loci, and the use of a cumulative risk-allele count (0–4) as a continuous predictor, which may offer greater sensitivity than the dominant model used here.

In conclusion, the presence of the CFH risk allele showed a preliminary association with lower photopic b-wave amplitudes, whereas no corresponding association was observed for ARMS2 genotypes. Given the small sample size and limited statistical power, this finding should be interpreted cautiously and requires confirmation in larger prospective controlled studies. Our findings support further prospective controlled studies investigating electrophysiological biomarkers and retinal resilience pathways in intermediate AMD.

## Figures and Tables

**Figure 1:**
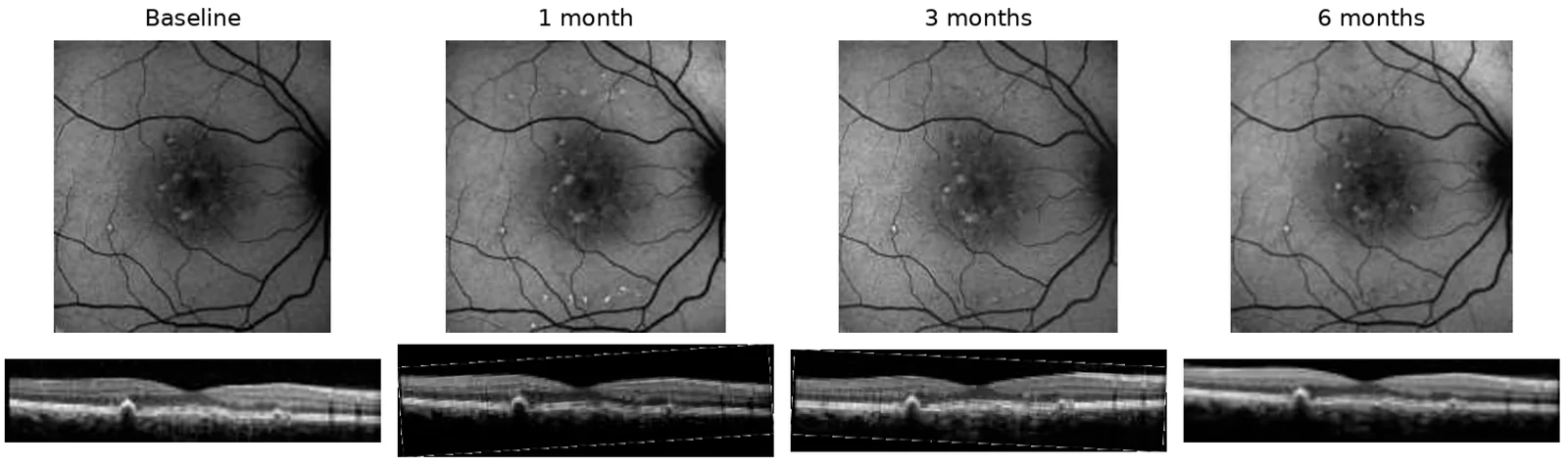
Multimodal imaging in a typical iAMD patient at baseline, 1, 3, and 6 months after 2RT laser. The 6-month acquisition is shown for illustrative purposes and was not included in the analysis. In the autofluorescence image (top row) the parafoveal hyperfluorescent spots indicate the drusen.

**Figure 2:**
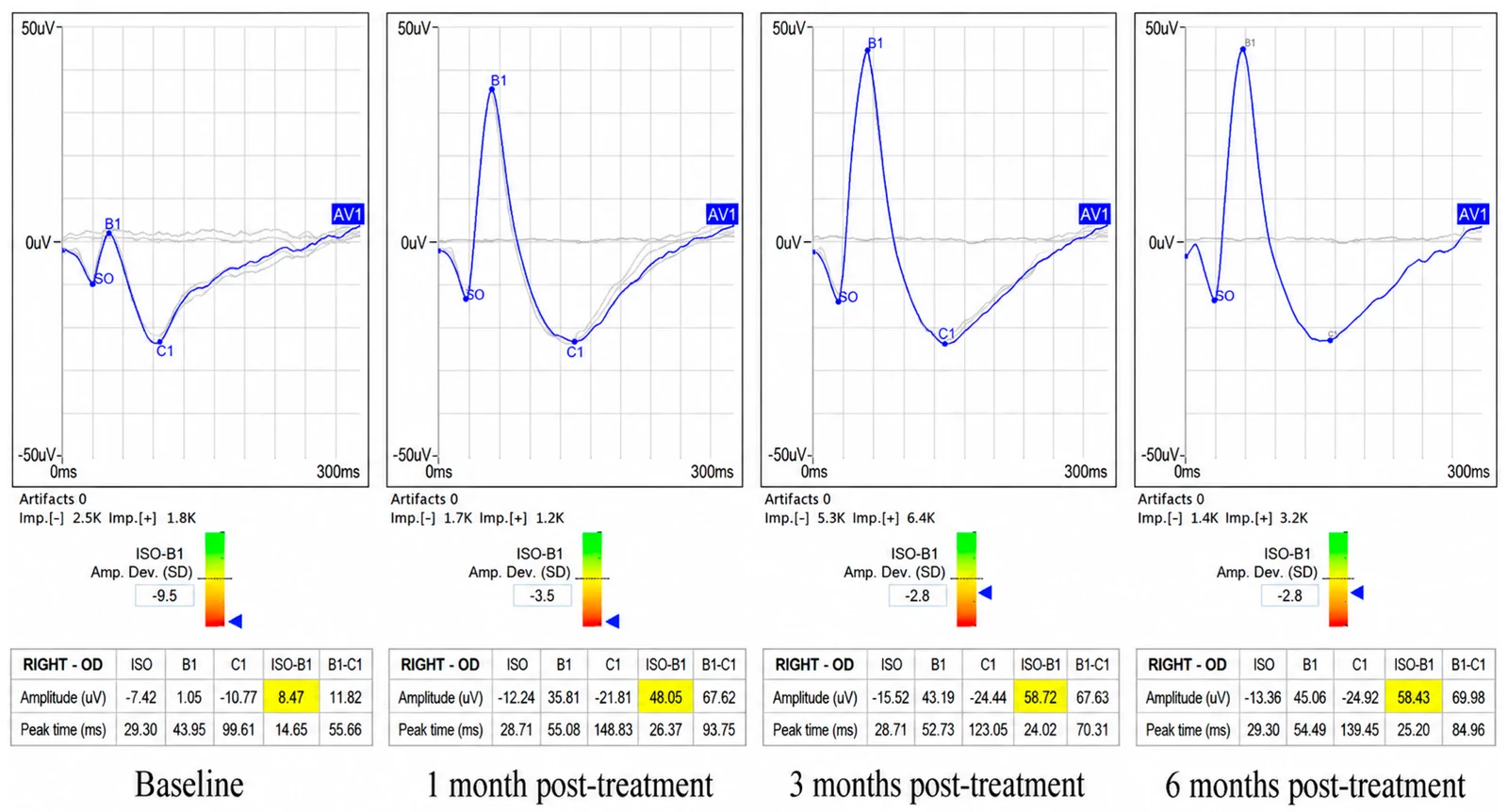
Individual example of mixed rod–cone ERG recordings obtained at baseline and at 1, 3, and 6 months after 2RT treatment. The 6-month recording is shown for illustrative purposes and was not included in the inferential longitudinal analysis.

**Figure 3:**
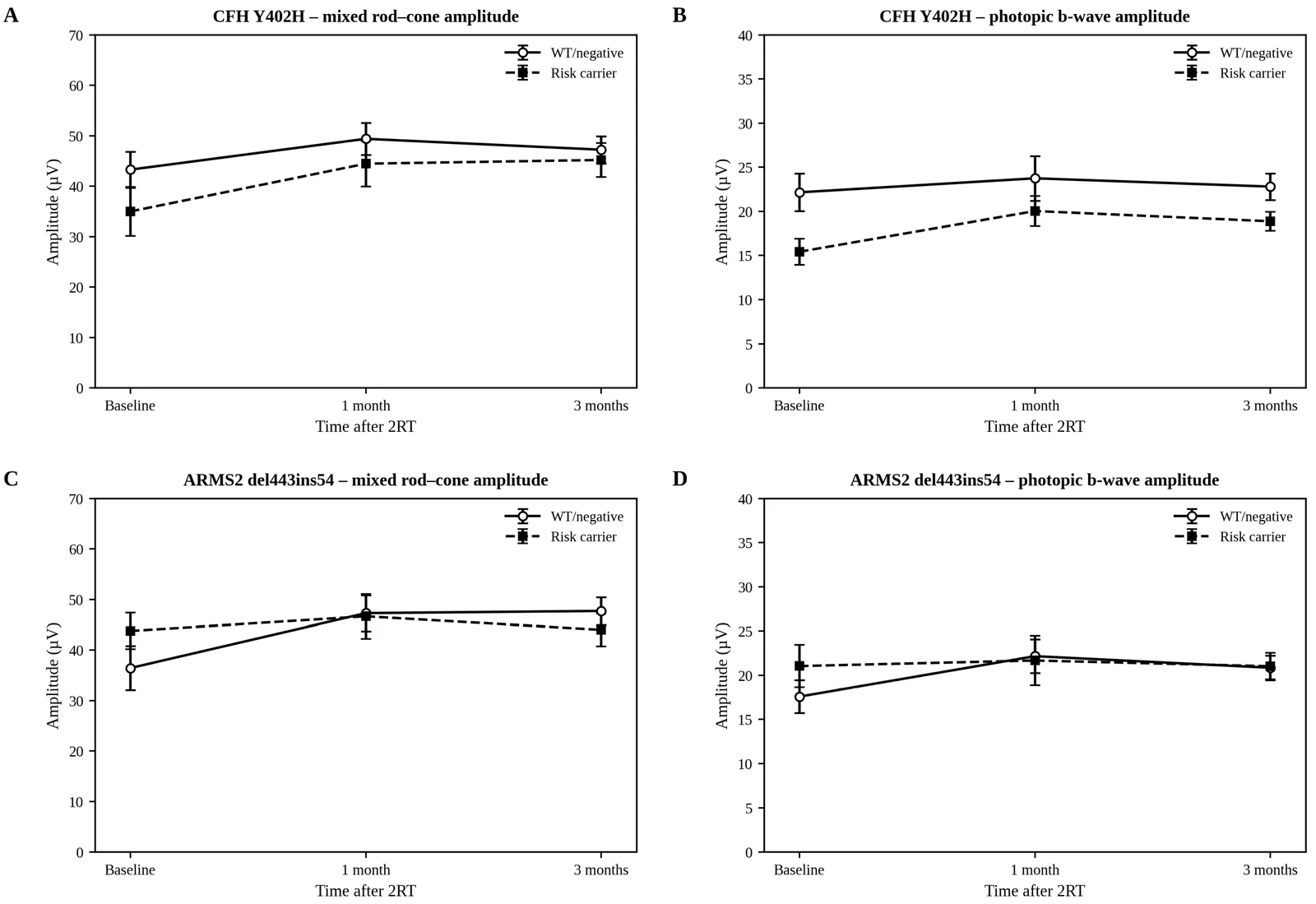
Full-field ERG changes during the 3-month follow-up after 2RT treatment. Mean ± SEM mixed rod–cone and photopic b-wave amplitudes are shown at baseline and at 1 and 3 months, separately for CFH and ARMS2 risk carriers and WT/negative patients.

**Table 1:** Summary of demographic, clinical and genetic characteristics of the study cohort.

Characteristic	Data
Number of patients	23
Age, years	72.8 ± 8.4 (range, 56–88)
Sex (male/female)	6/17
Ethnicity	Caucasian
Treated study eye (right/left)	15/8
Baseline BCVA, ETDRS letters	81.8 ± 3.4 (range, 76–85)
*CFH* (rs1061170, T/C)	WT/negative: 12; heterozygous for the risk allele: 7; homozygous for the risk allele: 4
*ARMS2* (rs10490924, G/T)	WT/negative: 14; heterozygous for the risk allele: 8; homozygous for the risk allele: 1
Risk-genotype distribution	Neither variant: 6; *CFH* only: 8; *ARMS2* only: 6; both variants: 3

**Table 2:** Normal ERG measurements in the control population.

Parameter	Mean (SEM)	95% Confidence Limit
Mixed rod–cone b-wave amplitude	50 (2.5)	30 (lower)
Mixed rod–cone b-wave implicit time	48 (1.3)	55 (upper)
Cone b-wave amplitude	26 (1)	15 (lower)
Cone b-wave implicit time	29 (1)	34 (upper)

## Data Availability

Data available on request due to restrictions (privacy reasons): the data presented in this study are available on request from the corresponding author.

## References

[1] Mitchell P., Liew G., Gopinath B., Wong T.Y. (2018). Age-related macular degeneration. Lancet.

[2] Fleckenstein M., Keenan T.D.L., Guymer R.H., Chakravarthy U., Schmitz-Valckenberg S., Klaver C.C., Wong W.T., Chew E.Y. (2021). Age-related macular degeneration. Nat. Rev. Dis. Primers.

[3] Curcio C.A., Johnson M., Ryan S.J., Sadda S.R., Hinton D.R., Schachat A.P., Sadda S.R., Wilkinson C.P., Wiedemann P. (2013). Structure, function, and pathology of Bruch’s membrane. Retina.

[4] Kim J., Lee Y.J., Won J.Y. (2021). Molecular Mechanisms of Retinal Pigment Epithelium Dysfunction in Age-Related Macular Degeneration. Int. J. Mol. Sci..

[5] Keenan T.D.L., Hébert M., Chew E.Y., Johnson M.W. (2026). What Should Patients with Age-Related Macular Degeneration Eat?. Am. J. Ophthalmol..

[6] Zong Y., Fan Q., Qiu S., Zhang H., Cen Z. (2026). The Molecular Basis of Ocular Aging: Mechanisms, Pathologies, and Emerging Therapeutics. Investig. Ophthalmol. Vis. Sci..

[7] Jabbarpoor Bonyadi M.H., Yaseri M., Nikkhah H., Bonyadi M., Soheilian M. (2018). Association of risk genotypes of ARMS2/LOC387715 A69S and CFH Y402H with age-related macular degeneration with and without reticular pseudodrusen: A meta-analysis. Acta Ophthalmol..

[8] Altankhuyag A., Ganbold C., Byambadorj B., Tumurbaatar S., Sodnomtseren P., Davaatseren U., Jav S. (2024). The interactions between ARMS2, CFH, VEGF-A and environmental factors on the risk of age-related macular degeneration. Mol. Vis..

[9] Vavvas D.G., Small K.W., Awh C.C., Zanke B.W., Tibshirani R., Kustra R. (2018). CFH and ARMS2 genetic risk determines progression to neovascular age-related macular degeneration after antioxidant and zinc supplementation. Proc. Natl. Acad. Sci. USA.

[10] Chu L., Bi C., Wang C., Zhou H. (2024). The Relationship between Complements and Age-Related Macular Degeneration and Its Pathoge-nesis. J. Ophthalmol..

[11] Jabbarpoor Bonyadi M.H., Yaseri M., Soheilian M. (2020). Association of combined complement factor H Y402H and ARMS2/LOC387715 A69S polymorphisms with age-related macular degeneration: An updated meta-analysis. Ophthalmic Genet..

[12] Farashi S., Abbott C.J., Ansell B.R.E., Wu Z., Altay L., Arnon E., Arnould L., Bagdasarova Y., Balaskas K., Chen F.K. (2025). HTRA1/lncRNA HTRA1-AS1 dominates in age-related macular degeneration reticular pseudodrusen genetic risk with no complement involvement. Nat. Commun..

[13] Swaroop A., Roska B. (2026). Revisiting retinal and macular degeneration in the genomics era. Nat. Rev. Genet..

[14] Fritsche L.G., Igl W., Bailey J.N.C., Grassmann F., Sengupta S., Bragg-Gresham J.L., Burdon K.P., Hebbring S.J., Wen C., Gorski M. (2016). A large genome-wide association study of age-related macular degeneration highlights contributions of rare and common variants. Nat. Genet..

[15] Lu F., Liu S., Hao Q., Liu L., Zhang J., Chen X., Hu W., Huang P. (2018). Association Between Complement Factor C2/C3/CFB/CFH Polymorphisms and Age-Related Macular Degeneration: A Meta-Analysis. Genet. Test. Mol. Biomark..

[16] Jaskoll S., Shwartz Y., Kramer A., Elbaz-Hayoun S., Rinsky B., Grunin M., Tiosano L., Levy J., Vofo B.N., Chowers I. (2025). Genetic Risk and OCT-Based Phenotypic Associations in Age-Related Macular Degeneration. Ophthalmol. Sci..

[17] Sardell R.J., Persad P.J., Pan S.S., Whitehead P., Adams L.D., Laux R.A., Fortun J.A., Brantley M.A., Kovach J.L., Schwartz S.G. (2016). Progression Rate from Intermediate to Advanced Age-Related Macular Degeneration Is Correlated with the Number of Risk Alleles at the CFH Locus. Investig. Ophthalmol. Vis. Sci..

[18] Fabrizio C., Termine A., Caputo V., Megalizzi D., Zampatti S., Falsini B., Cusumano A., Eandi C.M., Ricci F., Giardina E. (2022). WARE: Wet AMD risk-evaluation tool as a clinical decision-support system integrating genetic and non-genetic factors. J. Pers. Med..

[19] Hang A., Shao A., Shea M., Roux M.J., Imai-Leonard D.M., Adams D.J., Amano T., Amarie O.V., Berberovic Z., Bour R. (2025). Ocular Phenotyping of Knockout Mice Identifies Genes Associated with Late Adult Retinal Phenotypes. Investig. Ophthalmol. Vis. Sci..

[20] Ferris FL3rd Wilkinson C.P., Bird A., Chakravarthy U., Chew E., Csaky K., Sadda S.R. (2013). Clinical classification of age-related macular degeneration. Ophthalmology.

[21] Chew E.Y., Clemons T.E., Agrón E., Domalpally A., Keenan T.D.L., Vitale S., Weber C., Smith D.C., Christen W., AREDS2 Research Group (2022). Long-term Outcomes of Adding Lutein/Zeaxanthin and ω-3 Fatty Acids to the AREDS Supplements on Age-Related Macular Degeneration Progression: AREDS2 Report 28. JAMA Ophthalmol..

[22] Li Y., Andrew N., LaHood B.R. (2022). Evidence-based vitamin supplements for age-related macular degeneration: An analysis of available products. Clin. Exp. Optom..

[23] Merry G.F., Munk M.R., Dotson R.S., Walker M.G., Devenyi R.G. (2017). Photobiomodulation reduces drusen volume and improves visual acuity and contrast sensitivity in dry age-related macular degeneration. Acta Ophthalmol..

[24] Grewal M.K., Sivapathasuntharam C., Chandra S., Gurudas S., Chong V., Bird A., Jeffery G., Sivaprasad S. (2020). A pilot study evaluating the effects of 670 nm photobiomodulation in healthy ageing and age-related macular degeneration. J. Clin. Med..

[25] Jaffe G.J., Westby K., Csaky K.G., Mones J., Pearlman J.A., Patel S.S., Joondeph B.C., Randolph J., Masonson H., Rezaei K.A. (2021). C_5_ inhibitor avacincaptad pegol for geographic atrophy due to age-related macular degeneration: A randomized pivotal phase 2/3 trial. Ophthalmology.

[26] Liao D.S., Grossi F.V., El Mehdi D., Gerber M.R., Brown D.M., Heier J.S., Wykoff C.C., Singerman L.J., Abraham P., Grassmann F. (2020). Complement C_3_ inhibitor pegcetacoplan for geographic atrophy secondary to age-related macular degeneration: A randomized phase 2 trial. Ophthalmology.

[27] Guymer R.H., Brassington K.H., Dimitrov P., Makeyeva G., Plunkett M., Xia W., Chauhan D., Vingrys A., Luu C.D. (2014). Nanosecond-laser application in intermediate AMD: 12-month results of fundus appearance and macular function. Clin. Exp. Ophthalmol..

[28] Guymer R.H., Wu Z., Hodgson L.A.B., Caruso E., Brassington K.H., Tindill N., Aung K.Z., McGuinness M.B., Fletcher E.L., Chen F.K. (2019). Subthreshold nanosecond laser intervention in age-related macular degeneration: The LEAD randomized controlled clinical trial. Ophthalmology.

[29] Capoluongo E., Concolino P., Piccardi M., Marangoni D., Mello E., Minnella A.M., Savastano C., Fadda A., Zuppi C., Bisti S. (2012). Retinal function and CFH-ARMS2 polymorphisms analysis: A pilot study in Italian AMD patients. Neurobiol. Aging.

[30] Cascella R., Stocchi L., Strafella C., Mezzaroma I., Mannazzu M., Vullo V., Montella F., Parruti G., Borgiani P., Sangiuolo F. (2015). Comparative analysis between saliva and buccal swabs as source of DNA: Lesson from HLA-B*57:01 testing. Pharmacogenomics.

[31] Galli-Resta L., Falsini B., Rossi G., Piccardi M., Ziccardi L., Fadda A., Minnella A., Marangoni D., Placidi G., Campagna F. (2016). Bilateral Symmetry of Visual Function Loss in Cone-Rod Dystrophies. Investig. Ophthalmol. Vis. Sci..

[32] Cusumano A., Falsini B., D’Ambrosio M., D’Apolito F., Sebastiani J., Levialdi Ghiron J.H., Giardina E., Cascella R. (2023). Long-Term Structural and Functional Assessment of Doyne Honeycomb Retinal Dystrophy following Nanosecond 2RT Laser Treatment: A Case Series. Case Rep. Ophthalmol..

[33] Guymer R.H., Blair J.P.M., De Zanet S., Apostolopoulos S., Ciller C., Wu Z., LEAD Study Group (2025). Effect of Subthreshold Nanosecond Laser on Loss of OCT Outer Retinal Bands in Age-Related Macular Degeneration: A LEAD Study Report. Ophthalmol. Sci..

[34] Vessey K.A., Ho T., Jobling A.I., Wang A.Y., Greferath U., Fletcher E.L. (2018). Nanosecond laser treatment for age-related macular degeneration does not induce focal vision loss or new vessel growth in the retina. Investig. Ophthalmol. Vis. Sci..

[35] Wood J.P.M., Tahmasebi M., Casson R.J., Plunkett M., Chidlow G. (2021). Physiological response of the retinal pigmented epithelium to 3-ns pulse laser application, in vitro and in vivo. Clin. Exp. Ophthalmol..

[36] Stone J., Mitrofanis J., Johnstone D.M., Falsini B., Bisti S., Adam P., Nuevo A.B., George-Weinstein M., Mason R., Eells J. (2018). Acquired resilience: An evolved system of tissue protection in mammals. Dose Response.

